# 910 Trends in Pediatric Burn Incidence and Care: Comparative Analysis of Burn Centres and Non-specialized Facilities

**DOI:** 10.1093/jbcr/iraf019.441

**Published:** 2025-04-01

**Authors:** Eduardo Gus, Teresa To, Joel Fish, Christina Diong, Natasha Saunders

**Affiliations:** The Hospital for Sick Children; The Hospital for Sick Children; The Hospital for Sick Children; Institute for Clinical Evaluative Sciences; The Hospital for Sick Children

## Abstract

**Introduction:**

Burn centres are established to provide specialized care for burn patients, ensuring they receive the necessary intensity and expertise for the best outcomes. In our province, the volume of patients treated at specialized burn centres has increased, even though pediatric burn injury rates have declined in other high-income countries. It is unclear whether this increase reflects a rise in severe burn incidence in our province or changes in how burn care is delivered. We aimed to examine temporal trends in pediatric burn incidence, focusing on different types of hospitals (burn centres vs. non-specialized facilities) and target population (pediatric vs. adult/mixed population burn centres).

**Methods:**

This was a population-based repeated, cross-sectional study using linked health administrative datasets. Using emergency department and hospital records, we identified all children 0-17 years old with a hospital visit for burn injury between 2003-2022. Burn injury trends for burns treated at burn centres vs. non-specialized facilities and pediatric vs. adult/mixed population burn centres were tested with Poisson regression.

**Results:**

Over 20 years, pediatric burn injury incidence decreased by 37% (165/100,000 to 104/100,000 population; relative rate [RR] 0.98, 95% confidence interval [CI] 0.98-0.98). Burn injuries managed at non-specialized facilities decreased 45% (144/100,000 to 79/100,000 population; RR 0.97, 95% CI 0.97-0.97) while those managed at burn centres increased 20% (21/100,000 to 25/100,000 population; RR 1.02, 95% CI 1.01-1.03) over this same period. While pediatric burns treated at adult/mixed population burn centres decreased 36% (7.6/100,000 to 4.9/100,000; RR 0.98, 95% CI 0.97-0.98), those treated at stand-alone pediatric burn centres increased 51% (13/100,000 to 20/100,000; RR 1.03, 95% CI 1.03-1.04) over the study period.

**Conclusions:**

Pediatric burn incidence has declined over 20 years with a concomitant increased proportion managed at burn centres. Pediatric burn care has also shifted from adult/mixed population to stand-alone pediatric burn centres.

**Applicability of Research to Practice:**

Shifts in delivery of care over 20 years demonstrate increasing trends of pediatric burns being treated at burn centres. Given the observed changes, ensuring the intensity of burn services and expertise available match the needs of patients and their injuries is important for efficiency and health care quality.

**Funding for the Study:**

This study was funded by a Studentship Award.

